# The effect of female mating status on male offspring traits

**DOI:** 10.1007/s00265-014-1683-1

**Published:** 2014-01-31

**Authors:** D. Gottlieb, Y. Lubin, A. R. Harari

**Affiliations:** 1 Department of Life Sciences, Ben-Gurion University of the Negev, P.O. Box 653, Beer Sheva, 84105 Israel; 2 Mitrani Department of Desert Ecology, Blaustein Institutes for Desert Research, Ben-Gurion University of the Negev, Midreshet Ben-Gurion, Israel; 3 Department of Entomology, Agricultural Research Organization, the Volcani Center, Bet-Dagan, Israel; 4 Present Address: School of Biological Sciences, University of Bristol, Woodland Road, Bristol, BS8 1UG UK

**Keywords:** *Coccotrypes dactyliperda*, Local mate competition, Male dispersal, Oviposition, Trade-off, Unmated females

## Abstract

In haplodiploid insects, males develop from unfertilized eggs; consequently, unmated females can reproduce. In a patchy, highly structured population, where brothers compete for mates and the reproductive return through sons is lower, females should minimize the number of male offspring. Consequently, unmated females are likely to have a reduced fitness compared to mated females. Here, we tested the oviposition behaviour of the haplodiploid beetle *Coccotrypes dactyliperda*. In this species, the unmated female can mate with her son to produce daughters. We predicted that unmated females could increase their fitness by (1) producing only few and small sons sufficient for mother–son mating and (2) dispersing to a patch occupied by conspecific females in order to increase their or their sons’ chance of mating. We demonstrate that (1) unmated females are common (23 % of all females), (2) they oviposit more frequently than mated females in occupied patches, (3) unmated females oviposit more eggs than mated females—this is in spite of the trade-offs, evident in this study, between the number of sons and the number of the mother’s future offspring after mating, (4) unmated females have a higher proportion of dispersing sons, and (5) sons of unmated females are smaller than sons of mated females. We conclude that the incidence of unmated females in the structured populations of *C. dactyliperda* is explained by plasticity in their oviposition behaviour. We discuss conditions where a high incidence of unmated females can persist as a successful strategy in structured populations.

## Introduction

In most animal species, mating is essential for reproduction. This, however, is not necessarily the case in haplodiploid insects; females result from mating followed by fertilization of eggs (diploid), whereas males develop from unfertilized eggs (haploid) (Heimpel and de Boer 2008). Thus, unmated females are able to reproduce, although they will produce only male offspring. This in turn might lead to a reduction in their offspring success in encountering mating opportunities, thus incurring reproductive cost. The magnitude of this cost can affect the incidence of unmated females in the population and is strongly influenced by the structure of the population (reviewed in: Borsa and Kjellberg 1996; West and Herre 1998a; Hardy and Godfray 1990). In spatially structured populations, the offspring of one or a few mothers may mate amongst themselves in the natal patch before the females disperse. In such populations, a female-biased sex ratio is favoured due to “local mate competition” (LMC, Hamilton 1967). Under LMC, unmated females that produce several sons will have limited fitness because they are unable to produce the favoured female-biased sex ratio (e.g. Hardy and Godfray 1990; Godfray and Hardy 1993; and reviewed in: Hardy et al. 1998).

Theoretical and empirical studies suggest a selective advantage to mated females in highly inbred haplodiploid populations where females can control precisely the sex of their offspring (e.g. Green et al. 1982; Macke et al. 2012). However, the occurrence of a high frequency of unmated females in structured populations is not unusual for haplodiploids (e.g. beetles: 19 % in *Xylosandrus germanus* (Peer and Taborsky 2004); 30 % in *Xyleborinus saxesenii* (Biedermann 2010); non-pollinating wasps: 32 % in *Goniozus nephantidis* (Hardy and Cook 1995; West et al. 1997)). These examples suggest that the reduced mating success of unmated females’ sons might not be as high as expected. In moderate levels of population structure, mated females can reduce the cost of unmated females (Godfray 1990; Hardy and Godfray 1990; Ode et al. 1997). Interestingly, unmated females, under certain conditions, can also reduce the cost of producing male broods (e.g. Abe et al. 2010).

In certain haplodiploid species, the reduced fitness of unmated females may select for mother–son mating, i.e. unmated females produce males and then mate with at least one of them, to produce female offspring (*Mellitobia*: Balfour-Browne 1922; Borgia 1980; Adamson and Ludwig 1993; Abe et al. 2010). Ideally, due to the possible trade-off between current and future offspring (Godfray 1990), an unmated female should lay only one male egg. Nevertheless, under certain conditions, e.g. high male developmental mortality (Heimpel 1994; Nagelkerke and Hardy 1994) or under partial LMC (West and Herre 1998a), oviposition of more than one male is expected.

Developmental mortality of males can occur, i.e. equal developmental mortality of females, or can occur due to factors unique to males such as the expression of deleterious mutations in the haploid males (Smith and Shaw 1980) and sexual asymmetric susceptibility to competition and aggressive behaviour (e.g. Kapranas et al. 2011). As an insurance against premature death of sons, both unmated and mated females will benefit from increasing the number of males in the primary sex ratio within a clutch (West and Herre 1998b). By doing so, unmated females may increase their own mating probability, and mated females may minimize the probability of their daughters remaining unmated (West and Herre 1998b). However, the number of eggs a female can lay is often limited (Gottlieb et al. 2011), and thus females should not have more than a certain number of males per clutch because this may reduce the number of future female progeny. Given egg limitation, females may eventually produce only few sons, and mated females may end up having all-female clutches if male mortality is unpredictably high (Heimpel 1994; Hardy et al. 1998; Hardy and Mayhew 1998). As egg limitation decreases, i.e. females can produce larger clutch size, the probability of having all-female clutch decreases because the disadvantage of producing unmated daughters increases relative to the disadvantage of producing a son instead of a female (Green et al. 1982).

Females may benefit from ovipositing more than one son under partial LMC, for example, if males (Wrensch and Ebbert 1993; Greeff et al. 2003; Peer and Taborsky 2005) or unmated females (reviewed in Hardy 1994) disperse from the natal patch. In this system, the fitness of a dispersing male depends both on the population sex ratio, where male fitness increases when males are rare (reviewed in Hardy 1994), and on the probability of finding an occupied patch (West and Herre 1998a). In the latter case, mated females may produce males that are morphologically specialized for dispersal (e.g. fig wasps: Greeff 1995; Cook et al. 1997). Dispersal of unmated females in partial LMC, although common, does not necessarily increase their opportunities to mate. Only in rare cases does an unmated female find a patch occupied by another female with sons (Godfray 1990; West and Herre 1998a). Additionally, the unmated female could also lay eggs that will develop and subsequently mate with the females in the patch (Kapranas et al. 2008).

Interestingly, as is now known, full local mating structures (Hamilton 1967) are the exception whereas partial LMC is far more common (reviewed in Hardy 1994). In light of the abundance of partial LMC in natural systems, it is now important to reconsider previously studied systems in order to evaluate the level of LMC and its effect upon the reproductive fitness of unmated females. The aim of this study was to re-examine the mating system of the scolytid beetle *Coccotrypes dactyliperda*, previously considered a strict LMC system, through a detailed study of the behaviour of the unmated females.

The life history of this haplodiploid beetle is characterized by LMC (Gottlieb et al. 2011). *C. dactyliperda* develops in and feeds upon date palm seeds (*Phoenix* sp.), whose availability is restricted in time and space. Thus, suitable seed for oviposition may be scarce and may lead to a delay in oviposition (Gottlieb et al. 2011). Following Hamilton (1967) LMC model, the seed in this species seems to function as a patch: (1) A single date seed of *Phoenix datctylifera* (averaging 3 × 1 cm) provides all the required nutrients for the sequential development of four to five clutches, with an average of 30 individuals per clutch (Gottlieb et al. 2011). (2) Mature offspring mate in the seed and most females disperse to a new seed where they remain and oviposit their entire egg load. (3) Seed colonization usually takes place individually, but in high beetle densities, several females may colonize a single seed (Gottlieb et al. 2009). (4) Ovipositing females respond to the relatedness of other females colonizing the same date by adjusting their offspring sex ratio (Gottlieb et al. 2011). (5) Males usually remain in the natal seed.

The beetles’ reproductive season is limited to 6 months, during which time up to five generations can develop. The female is synovigenic, and eggs are produced continuously throughout her lifetime (Bar-Shalom and Mendel 2003; Zchori-Fein et al. 2006). Male eggs are oviposited and develop (18–21 days from egg to adult) before females (21–24 days). Males have reduced, non-functional flight wings (Herfs 1950; Sitkov-Sharon et al. 2010). *C. dactyliperda*’*s* life history strongly suggests that it is likely that females in the wild have the opportunity to mate with their sons. Adult female’s lifespan can get up to 58 days (Gottlieb et al. 2011); thus, an overlap between generations is the rule (Herfs 1950; Sitkov-Sharon et al. 2010), following with an extreme level of inbreeding (Gottlieb et al. 2009), and males are rarely found alone in a seed (DG personal observations). There is no current information on the frequency of mother–son mating in the wild. However, such mating even at a low frequency can cause significant reproductive gain to the unmated female (Borgia 1980). Thus, taking into account mother–son mating, we examine the effect of female mating status on male offspring traits. We predicted the following: (1) Under partial LMC, unmated females will increase their fitness by producing males that will disperse by walking from the natal patch. (2) The size of male offspring should depend on their mother’s mating status (mated or unmated). Since size in insects often correlates with developmental time and with reproductive success (e.g. Honek 1993; Harari et al. 1999; Mayhew and Glaizot 2001; Gottlieb et al. 2011), unmated females will benefit from sons with short development time, albeit at the cost of a decrease in their size and thus their future reproductive success. In contrast, due to competition, sons of mated females that will mate with their female siblings or additional same-patch neighbouring females (Gottlieb et al. 2010) will benefit from being large (Balfour-Browne 1922). (3) Given the choice between fresh (i.e. intact, unoccupied seeds) and occupied seeds, unmated females that seek mating opportunities will preferentially oviposit in the latter, whereas mated females should inhabit unoccupied seeds to reduce offspring competition over food. (4) Unmated females will preferentially oviposit in seeds occupied by other unmated females (a) to reduce possible competition over food in the seed, as unmated females are likely to have fewer offspring than mated females, and (b) to speed up their waiting time for a mate.

## Methods

Date seeds were collected in March 2007 under *P. dactylifera* date trees in Sha’ar Hagolan, northern Israel (N 32°41′11.4″, E 35°36′11.87″), and examined for the presence of *C. dactyliperda*. Seeds with beetles were taken to the laboratory where a colony was established at 28 °C and 12:12 L:D. Fresh seeds were collected from trees in the Arava Valley, an uninfected site in southern Israel, and were frozen until required. Experiments were carried out after rearing the beetles in the laboratory with unlimited amount of seeds for four generations.

To measure size and determine the sex of the offspring, we opened seeds inhabited by a single female. Offspring’s sex was determined by scrutinizing the distinct differences in colour and eye size of male and females (following Herfs 1950). We measured the adult’s head width as an index of size, as it is known to correlate with body and wing length (Gottlieb et al. 2011). Opened seeds allow the survival of pupae and adults only. Since the sclerotized head capsule of the pupa mainly develops at the different stages of instars and is a good predictor for the shape and size of the adult head (Chapman 1969), we collected all pupae and provided them with conditions for their development into adults. Each pupa was kept in an Eppendorf tube (1.5 ml) with the same range of high humidity (70–80 % RH) and temperature (28°) levels as before (i.e. the conditions that the seeds were kept). Thus, sex ratio and size were determined for pupae after they emerged as adults and for adults present at the time of seed opening. We numbered the seeds and opened them randomly, revealing the serial number only after measuring the offspring. Measurements of the adults were taken after all beetles had been stored in the same humidity and temperature conditions for the same period. Head width was measured using a binocular dissecting microscope to the nearest 0.001 mm (Gottlieb et al. 2010) with the image capture software ProgRes® CapturePro (Jenoptik Laser Optic System, GmbH, Jena, Germany). For accurate measuring, we pressed the beetle into elastic material so that both corners of the head, characterized by five hairs, could be observed.

### Experimental design

#### The frequency of unmated females

Unmated females were allowed to mate with their brothers before being presented with a fresh seed (*N* = 212). After 21 days, we collected all mature offspring and pupae and estimated the clutch size and sex ratio (Gottlieb et al. 2011). We assume that all-female clutches, i.e. lacking male offspring, is a good proxy for the frequency of unmated females (West et al. 1998).

We used the generalized linear model (GLM) for binomially distributed data (binary logistic) to estimate the effect of clutch size (covariate) on the frequency of all-female clutches (1 vs. 0).

#### Offspring characteristics

We tested whether the female mating status (mated or unmated) affects the characteristics of her offspring. To obtain unmated females, we collected one pupa from each clutch and placed each pupa in 1.5-ml Eppendorf tubes. Upon emergence, we collected only the females, randomly picked half of them, and presented these with an excess of males to ensure mating, while the other half remained unmated. Each female of both treatments was provided with a sliced date seed (<2 mm in width) as food. These slices are sufficient for feeding but not for oviposition (Gottlieb et al. 2011). The following day, we removed all males, and each female (unmated *N* = 81; mated *N* = 37) was placed individually in a Petri dish along with an intact seed. Twenty-one days later, the approximate period of time for sons to mature and mate with their unmated mother (Gottlieb et al. 2011), the seeds of both treatments were opened, mothers and all offspring found inside and outside the seed were counted, and mature stages were measured (see details above). To estimate the cost of ovipositing before mating on the number and size of offspring after mating, each mother from the “unmated” treatment was separated from her sons (females are darker, larger, and with larger eyes) and was provided with a fresh whole seed (this procedure partially simulates the natural behaviour of the female beetle (Gottlieb, unpublished data). After an additional period of 21 days, we opened the seeds. At the time of opening, the second clutch had larvae in various stages of development with yet no pupae or adults, which might have been a result of seasonal variation in development time. Consequently, offspring body size was not measured for the second clutch (see Methods).

We used the generalized linear model/mixed model (GLM or GLMM) for log-link distribution (1) to test the effect of treatment, i.e. the state of the female (factor), on size of the first clutch 21 days after the onset of the experiment, and on number of sons, (2) to assess whether the mating status (mated or unmated as a factor), the sons’ location inside or outside patch (factors), and the clutch size (potential competition over food, as a covariate) predict the size of the sons, and (3) to determine whether the state of the females (factor) and number of sons (possible competition, as a covariate) have an effect on male dispersal, i.e. the proportion of males located outside the patch, and finally, (4) to estimate the effect that the number and size of offspring (covariates) before mating have on clutch size after mating, using the size of unmated females as a random factor.

#### Oviposition site

Unmated females that disperse to a patch occupied by conspecific females may benefit by increasing their or their sons’ chances of mating. Thus, we tested whether females have a preference for oviposition site (intact or occupied seed) taking into account their own mating status and that of the resident females. We allowed the resident female (mated or unmated) to enter a seed and oviposit for 21 days before a second female, the intruder, was added to the Petri dish, which contained both an intact and the occupied seed. We introduced the seeds to the females in a full-factorial design with two levels of intruder’s state (mated and unmated) and two levels of resident female’s state (mated and unmated). The site at which each of the females penetrated the patch was marked 5 days after introducing each female (resident and intruder) to the patch. We used the generalized linear model (GLM) for binomially distributed data (binary logistic) to estimate whether the female’s state (mated or unmated and resident or intruder) had an effect on her decision where to enter the seed and oviposit (new seed or occupied seed).

## Results

### The frequency of unmated females

**Fig. 1:**
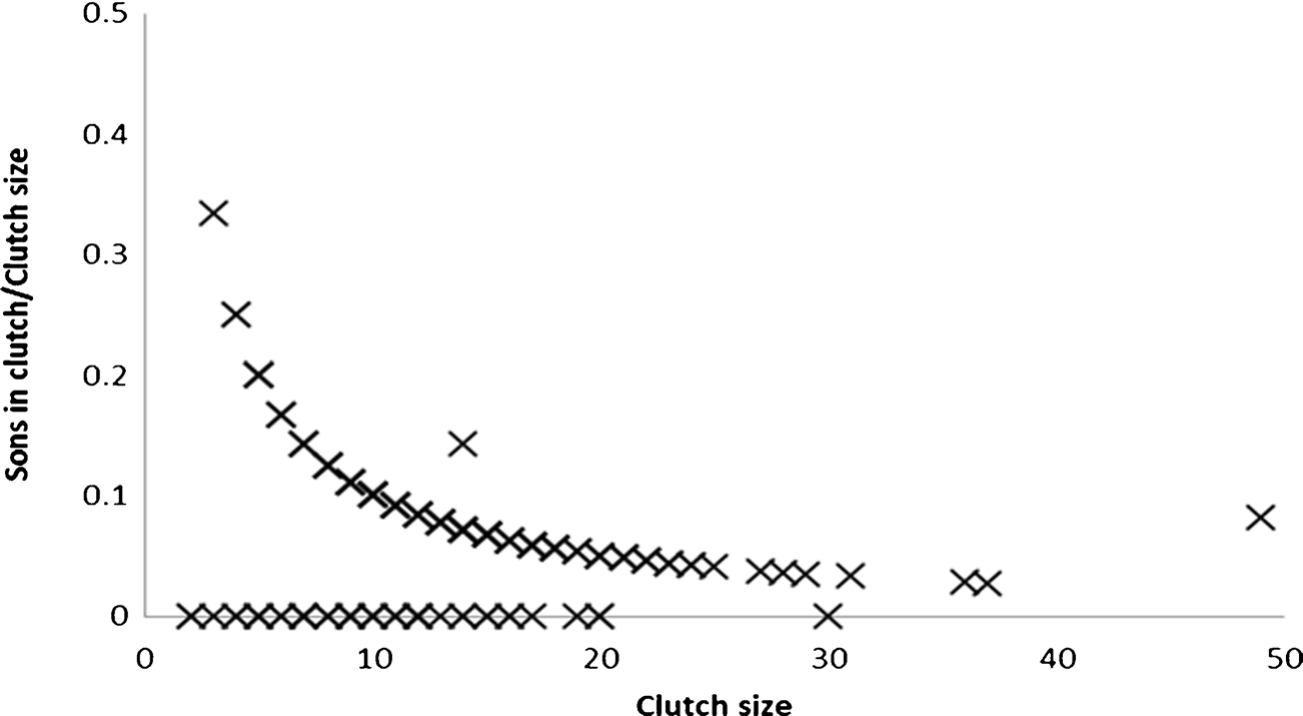
The proportion of sons in clutches of different sizes (*N* = 212, mated female clutches; logarithmic trend line, *R* ^2^ = 0.062). All clutches originate from mated females

Mated females (*N* = 212) produced between 0 and 4 sons per clutch (0 males, 23.23 %, 1 male, 75.83 %). In only two cases, there was more than one male (two males, 0.47 %, and four males, 0.47 %). Thus, we treated the response variable as binary (with or without males in the clutch). We used a generalized linear model with correction for overdispersion (using once the ratio of the deviance goodness of fit measure to its degrees of freedom and once adding a random effect). Sons were present significantly more in large clutches (binary logistic, *N* = 212, *x* ^2^ = 16.593, df = 1, *p* < 0.0001, Fig. 1).

### Offspring characteristics

#### Clutch size

**Fig. 2:**
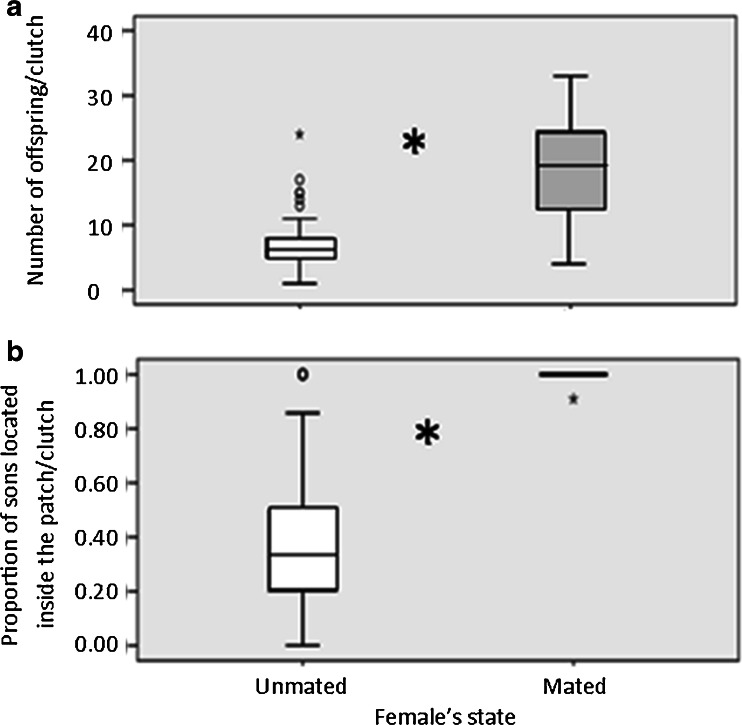
Number of offspring per clutch (**a**) and proportion of sons located inside the patch (**b**). Offspring of mated (*gray*) and unmated (*white*) mothers. *Large asterisk* indicates significant difference at the 5 % level. Each box plot represents the medians and 25th and 75th percentiles. Whiskers depict the values within 1.5 times the interquartile range. Extreme outliers are denoted by *circles* (see text for more details)

Mated females had a significantly larger clutch size (*N* = 40, median clutch size: 19.5, interquartile range (IQR): 12.50) than unmated females (*N* = 81, median: 6, IQR: 3, logit regression, *N* = 121, *x* ^2^ = 23.37, df = 1, *p* < 0.0001, Fig. 2a). However, mated females had fewer sons (*N* = 40, median number of males: 1, IQR: 0) than unmated females (*N* = 81, median number of males: 6, IQR: 3).

#### Proportion of sons inside the seed

The proportion of sons (out of all sons) of mated females that were found inside the seed was significantly higher (*N* = 40 seeds, median: 1, IQR: 0, Fig. 2b) than the proportion of sons inside the seed of unmated females (*N* = 81 seeds, median: 0.33, IQR: 0.30, logistic regression, *N* = 121 seeds, *x* ^2^ = 4.361, df = 1, *p* = 0.037), with the number of males in the clutch having no effect (logistic regression, *N* = 121 seeds *x* ^2^ = 0.0029, df = 1, *p* = 0.864) and with no interaction between the female’s state and the number of males in the clutch (logistic regression, *N* = 121, *x* ^2^ = 0.005, df = 1, *p* = 0.544).

#### Size of sons

**Fig. 3:**
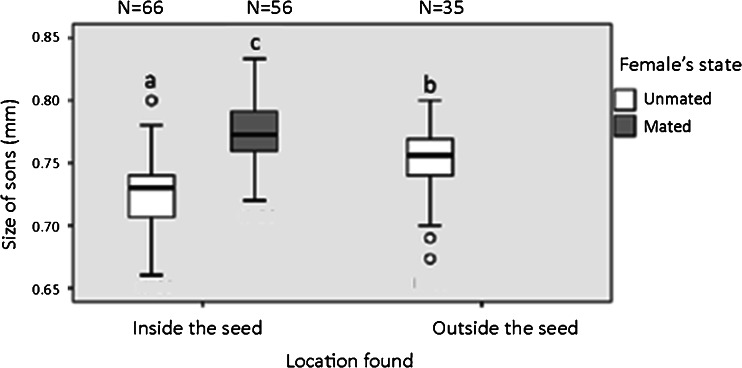
Size of males originating from unmated (*white*) and mated (*grey*) females. Non- overlapping letter sequences indicate significant difference at the 5 % significant level. Each box plot represents the medians and 25th and 75th percentiles. Whiskers depict the values within 1.5 times the interquartile range. Extreme outliers are denoted by *circles* (see text for more details)

Not all offspring were measured; this is due both to the destructive procedure of seed opening and the early developmental stages of some offspring (see Methods). Furthermore, since there was only one incident in which a son of a mated female was found outside the seed, we did not compare its size. Mating status of females had a significant effect on the location of their sons (inside and outside the seed) and the size of sons (logit regression, *N* = 157, *x* ^2^ = 15.018, df = 2, *p* < 0.001, *N* = 157, *x* ^2^ = 11.025, df = 2, *p* < 0.001, respectively, Fig. 3), while clutch size (logit regression, *N* = 157, *x* ^2^ = 0.054, df = 1, *p* < 0.816) and the interaction between mating status and clutch size (logit regression, *N* = 157, *x* ^2^ = 0.671, df = 2, *p* < 0.715) had no significant effect on the size of sons. Pairwise comparisons within the test showed that sons of unmated females located in the seed were significantly smaller (*N* = 66 seeds, median: 0.73 mm, IQR: 0.04) than those outside the seed (*N* = 56, median: 0.75 mm, IQR: 0.03) and that both were significantly smaller (*N* = 122, median: 0.74 mm, IQR: 0.03) than sons of mated females found in the seeds only (*N* = 35, median: 0.77 mm, IQR: 0.03, Fig. 3).

#### Potential cost of ovipositing sons

Thirty out of 81 females died before invading the second seed or before oviposition occurred. Both, number (*N* = 51, median: 6, IQR: 3) and body size (*N* = 51, median: 0.74, IQR: 0.13) of sons from unmated females were significantly negatively correlated with the number of offspring produced after the females had mated (*N* = 51, median: 27.00, IQR: 12.00, logit regression, *N* = 51, *x* ^2^ = 23.97, df = 1, *p* < 0.0001). Although mating was observed in all cases, we cannot be sure that all had been inseminated since when we opened the seeds, all offspring had not developed to pupal or adult stage. However, since clutch sizes were similar to the normal clutch size of mated females (around 30 offspring), we assumed that all females were fertilized.

#### Oviposition site

**Fig. 4:**
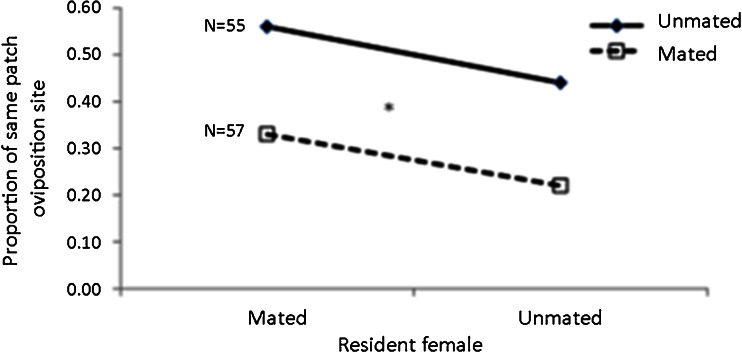
Proportion of same patch oviposition site for unmated (*bold line*) and mated (*dashed line*) intruders when resident females were mated or unmated. *Large asterisk* indicate significant difference at the 5 % significant level (see text for more details)

The state of the intruder females had a significant effect on their decision where to oviposit (*N* = 112, *x* ^2^ = 5.877, df = 1, *p* = 0.015, Fig. 4). Unmated females entered more occupied seeds (*N* = 55, 50 %) than the mated ones (*N* = 57, 28 %). Mating status of the resident female (mated or unmated) had no effect on the proportion of females invading the seed (*N* = 112, *x* ^2^ = 1.725, df = 1, *p* = 0.189, Fig. 4). No interaction was detected between intruder’s state and resident’s state (*N* = 112, *x* ^2^ = 0.004, df = 1, *p* = 0.951).

## Discussion

In agreement with the prediction, this study demonstrates that the high incidence of unmated females in structured populations (~23 % in this study) can be explained by the behavioural and developmental plasticity in *C. dactyliperda*. We show that the unmated females differ from the mated ones in a broad range of oviposition strategies: (1) Their sons are significantly smaller and (2) may disperse from their natal seeds. (3) Mated females oviposit more in unoccupied, intact seeds than did unmated females. We discuss conditions where a high incidence of unmated females can persist as a successful strategy in structured populations.

In a previous study (Gottlieb et al. 2010), the population of *C. dactyliperda* was described as highly structured, i.e. under strict LMC. As in other studies of LMC, Gottlieb et al. (2010) inferred the mating system of an organism from its population sex ratio (e.g. Pickering et al. 2000; West et al. 2000a, b). However, in his seminal study, Greeff (2002) demonstrated that sex ratio is a poor predictor of a mating system since it can obscure behaviours such as male dispersal. Following Greeff (2002) critique, we studied the oviposition behaviour of *C. dactyliperda*. We found that both males and unmated females can disperse from their natal patch, i.e. the population is not highly structured but rather is under partial LMC.

### Possible benefits of an all-female clutch

We found that the number of sons produced by unmated females negatively affects the number of offspring (daughters and sons alike) they may have after finally mating with their sons. This cost can be an outcome of the common trade-off between current vs. future reproduction of inbreeding depression caused by mother–son mating (e.g. Henter 2003) or of mating delayed by waiting for the son to mature (e.g. Gottlieb et al. 2011). Regardless of the factors contributing to the cost, the high incidence of all-female clutches suggests that this cost is partially or completely offset by other factors. For example, outbreeding, which is enabled by partial LMC (West and Herre 1998a), and the benefits of helping in the patch may offset the cost of ovipositing a surplus of sons in a small clutch as an insurance against high male mortality (Heimpel 1994; Nagelkerke and Hardy 1994).

Given the opportunity, *C. dactyliperda* females are inclined to outbreed rather than inbreed (Gottlieb et al. 2009). This does not come as a surprise, since outbreeding may lead to heterosis (Peer and Taborsky 2005). If females from all-female clutches manage to mate after dispersal, ovipositing all-female clutches may increase outbreeding probability, thereby promoting their own and their mother’s fitness. As *C. dactyliperda* are highly inbred, but with rare events of outbreeding (Gottlieb et al. 2010), heterosis is a less likely explanation for the high incidence of all-female clutches.

Increasing inclusive fitness through all-female clutches is a selective process that seemingly opposes outbreeding. The combination of sequential oviposition and the development of all-female clutches can lead to overlapping generations (haystack model) and can favour cooperative behaviour (reviewed in Abe et al. 2009). There is evidence for cooperation in *C. dactyliperda* life history: (1) more females from inbred populations remain in their natal seed and (2) produce a larger mean of larvae than females of outbreeding populations (Sitkov-Sharon et al. 2010). Cooperation in *C. dactyliperda* life history could further induce females originating from small all-female clutches to remain in the patch and gain inclusive fitness through taking care of their new born sisters.

The primitively eusocial beetle, *X. saxesenii*, has some life history traits in common with *C. dactyliperda*: high genetic relatedness (*X. saxesenii*: Peer and Taborsky 2007, *C. dactyliperda*: Gottlieb et al. 2009) and timing of dispersal, which depends on the condition within the nest, gallery protection, delayed dispersal, and overlapping generations (*X. saxesenii*: Biedermann and Taborsky 2011, *C. dactyliperda*: Sitkov-Sharon et al. 2010). The similarities between *C. dactyliperda* and a species with cooperative behaviour (Kent and Simpson 1992; Kirkendall 1993; Jordal et al. 2002) support a possible cooperation among *C. dactyliperda* as well (Sitkov-Sharon et al. 2010).

All-female clutches are unlikely to occur by chance, or by sperm depletion, since all males in this system are oviposited at the start of the oviposition period (protandry, Herfs 1950) and mothers lay several clutches during their lifetime, reaching an average of 50 diploid female offspring in total (Gottlieb et al. 2011). Differential developmental mortality of offspring in a haplodiploid system may be the mechanism underlying the development of all-female clutches in *C. dactyliperda*, mainly in small clutches. Developmental mortality of males can occur due to the expression of deleterious mutations in haploid males (Smith and Shaw 1980) and due to sexual asymmetric susceptibility to competition (e.g. Kapranas et al. 2011). There is no evidence for directly aggressive male–male behaviour (personal observation, D. Gottlieb). Nevertheless, the possibility that offspring manipulate the sex ratio within the patch (as for example in polyembryonic parasitoid wasps; Ode and Strand 1995) should be further examined.

Further studies of this species are needed to clarify the role of secondary sex ratio disorders resulting in male mortality as a cause for the high frequency of all-female clutches (Khidr et al. 2013). If there are no secondary sex ratio disorders and the mother beetle from the very beginning controls the production of males, then either the chances for mating with non-related partners (and thus outbreeding) must be higher than observed (Biedermann 2010) or the cost of ovipositing a male must be higher than previously expected. Assessing fitness reduction in unmated females in the field, rather than in controlled conditions in the lab, may clarify the latter.

### Offspring characteristics

The results of this study indicate that unmated females allocate resources to their male offspring for two distinct tasks: mother–son mating and dispersal from the natal patch. Under LMC, mother–son mating can allow females to produce a subsequent mixed sex clutch that may bring substantially greater fitness returns than a large all-male clutch (Adamson and Ludwig 1993). As is evident in this and previous studies (Godfray 1990), there is a trade-off between the number of sons a female has before mating and the number of offspring (daughters and sons) she may produce after mating. Further studies, comparing between the trade-off of mated and unmated females will reveal whether the trade-off is an outcome of late mating and/or egg limitation. In both cases, ideally an unmated female should lay only one male egg and save the rest of her eggs to produce daughters. However, the results of this study support previous studies (e.g. Nagelkerke d Hardy 1994) demonstrating that unmated females oviposited more than one male per clutch (median: 6, IQR: 3). This suggests that the cost of having more than one male when unmated is outweighed by the benefits of other factors, e.g. insurance against the possibility that all sons die before maturity and of having more sons that will mate after dispersal.

The success rate of mother–son mating in various taxa is low (Hardy et al. 1999). Not only can sons die before mother–son mating can occur but the mother may also die prematurely. In this study, 37 % of the unmated females died after mating with their own son and before laying another clutch. This high mortality rate can be an outcome of aging; an unmated female will thus benefit from sons that mature fast, reducing the risk of maternal death before mating and cutting the time before producing daughters. It is well known that the rate of development is profoundly influenced by the amount and quality of resources allocated to offspring by mothers (reviewed in: Fox et al. 1997). This can affect offspring adult size, e.g. low load of yolk can lead to smaller size (reviewed in: Fox et al. 1997) and can explain the small size of sons of unmated females, found in this study (although, not all offspring were measured, see Methods). Additionally, offspring size can be an outcome of environmental constraint such as male–male aggressive behaviour (Kapranas et al. 2011) or competition over food. In this study, small males are not likely to be the outcome of competition for food since sons of mated females are larger than sons of unmated females even though the former have larger clutch sizes in otherwise a similar environment. Competition asymmetry favouring male competitors over females is also not likely as males are significantly smaller than females. Both effects, maternal and environmental effects, on male developmental rate and body size are currently being explored.

High mortality rate of unmated females can also be an outcome of suboptimal conditions outside the seed and/or the energetic cost of penetrating to a new seed. Both possibilities can indicate that dispersal in this species can be costly. Nevertheless, dispersal may be beneficial since it can increase the probability of encountering mating opportunities outside the natal patch. Thus, having dispersing sons may increase the fitness of unmated females. While female dispersal is almost an integral part of their life cycle, male *C. dactyliperda* have reduced flight wings (Herfs 1950). Hence, the males are not expected to disperse far from their natal patch. Nevertheless, from this study, it is evident that sons of unmated females do disperse as a high proportion of them were found walking outside their natal patch. These sons were significantly larger than sons that remained within the patch (both having the typical reduced flying wings). Large male offspring may have increased endurance in dispersal and increased success in intra-male competition for females (Okada et al. 2007) in a new patch. However, having large sons can lead to an increase in developmental time and a decrease in the number of next clutch offspring. These contrasting benefits and costs can explain why dispersing sons of unmated females are still significantly smaller than those of mated females.

Unmated *C. dactyliperda* females seem to overcome the cost of their sons’ dispersal and the need to increase their outbreeding success by preferring to oviposit significantly more than mated females in previously occupied patches. Although a fresh patch offers less competition for food and more oviposition sites, occupied seeds offer the females instant mates. When the seed does not provide mates, e.g. if the resident female has an all-female clutch, it nevertheless can provide future mating opportunities for their male offspring. Oviposition by an unmated female in a seed occupied by a mated female may lead to strong indirect competition over resources available in the seed (personal observation, D. Gottlieb). However, in contrast to our predictions, the results of this study indicate that both mated and unmated females do not choose to oviposit more in sites occupied by an unmated female. Thus, although this study indicates that *C. dactyliperda* females can assess their own mating status and act accordingly, it might be that due to the rareness of unmated females outside their natal seed, the ability to assess the status of other females has not evolved.

To conclude, it is considered that in a structured population, where the reproductive return of males is lower, females should minimize the number of male offspring. However, our data contribute to a growing body of evidence for a high incidence of unmated females in structured populations (e.g. Heimpel 1994; West et al. 1997). This, as in previous studies, can be explained by the maintenance of females’ behavioural plasticity. In this study, we show that oviposition behaviour and offspring traits depends on female’s mating status: (1) Unmated females oviposit more frequently than mated females in occupied patches. (2) Unmated females oviposit more but have smaller, male offspring than mated females. This is in spite of the trade-offs, evident in this study, between number and size of sons and the number of the mother’s future offspring after mating. (3) Unmated females have a higher proportion of dispersing sons. Furthermore, this study increases our understanding of the various selective factors that might play a role in determining male body size; while there is sufficient evidence for selection favouring larger body size in male insects (e.g. Borgia 1982; Ward 1983; Carroll and Salamon 1995), evidence for selection favouring small body size is scarce (Schneider et al. 2000). We show that the size of male *C. dactyliperda* beetles can be adjusted phenotypically in accordance with their reproductive tasks. Small males mate with their mother (also evident in *Mellitobia*: Balfour-Browne 1922), while larger males originating from unmated females are destined to disperse and increase their mother’s reproductive success via outbreeding, and males originating from mated females are destined to inseminate their many sisters in the seed. Interestingly, although sons of mated females do not disperse at all, the IQR of the sizes of sons originating from both mated and unmated females are similar, suggesting that there is no strong selection for male dimorphism.
